# Positive Aspects of Caregiving in Incident and Long-Term Caregivers: Role of Social Engagement and Distress

**DOI:** 10.1093/geroni/igab046.978

**Published:** 2021-12-17

**Authors:** Chelsea Liu, Victoria Marino, Virginia Howard, William Haley, David Roth

**Affiliations:** 1 Harvard School of Public Health, Allston, Massachusetts, United States; 2 University of South Florida, Tampa, Florida, United States; 3 University of Alabama at Birmingham, Birmingham, Alabama, United States; 4 Johns Hopkins University, Baltimore, Maryland, United States

## Abstract

Positive aspects of caregiving (PAC) are positive appraisals that caregivers report about their role such as feeling appreciated or important, and may increase with caregiver adaptation over time. We aimed to examine differences in PAC by caregiving duration and social engagement, controlling for measures of distress. A total of 283 African American or White caregivers from the Caregiving Transitions Study with a wide range of caregiving durations were included in our analysis. We used multivariable linear regressions to model total PAC score on years of caregiving and social engagement (social network, monthly social contact), adjusting for age, sex, race, marital status, relationship to care recipient, dementia status of care recipient and measures of distress (depressive symptoms, perceived stress, caregiving strain). Caregivers with higher social engagement reported significantly higher PAC while caregivers with longer duration of care reported marginally higher PAC in most analytic models. African American caregivers reported higher PAC compared to White caregivers. Dementia caregivers reported lower PAC than non-dementia caregivers in models that adjusted for demographic variables and social network size, but the association was attenuated with the addition of caregiving strain. In summary, higher social engagement and longer care duration were associated with higher PAC after adjusting for demographic variables and measures of distress. Future studies should aim to understand how caregivers shift appraisal to positive aspects of their role and explore implementation of interventions targeting PAC in order to improve the caregiving experience.

